# Warning Signs Observed in Tanning Salons in New York City: Implications for Skin Cancer Prevention

**Published:** 2011-06-15

**Authors:** Corey H. Brouse, Charles E. Basch, Alfred I. Neugut

**Affiliations:** Columbia University; Columbia University, New York, New York; Columbia University, New York, New York

## Abstract

Use of artificial tanning may be contributing to the increased incidence of skin cancer. Federal law requires warning signs to inform consumers about health risks. All of the tanning facilities in New York City were assessed for compliance with this law during April and May 2010. More than one-third of the 224 tanning machines observed in 47 of the 85 facilities visited did not have any warning signs posted, and signs were difficult to see in many others.

## Objective

Skin cancer is the most common form of cancer in the United States, and rates of melanoma, the most dangerous form, are increasing (1). A main cause of skin cancer is ultraviolet radiation. Despite the known relationship between use of tanning machines and risk of basal and squamous cell carcinomas and melanoma (2) and that radiation from "tanning beds" has been deemed a carcinogen (3), use of indoor tanning has increased (4) and may be contributing to increased incidence of skin cancer. Several studies suggest that young girls (5) and adolescents (5) are most likely to engage in tanning, but high rates of tanning have been found in adults (6). Given that ultraviolet radiation is a known carcinogen (7), a clearly visible warning sign on each tanning machine is required by the US Food and Drug Administration (8) (Box). The warning is intended to alert the potential user about the danger of overexposure to ultraviolet radiation, of not using protective eyewear, and potential adverse interactions with certain cosmetics and medications.

Research on compliance with various indoor tanning regulations is limited, but studies suggest low compliance with posting the regulations (9-11). This noncompliance may impede informed decision making by consumers and pose a threat to public health. This study was conducted to estimate the prevalence of warnings in all of the tanning facilities in New York City.

**Box. Label Requirements and Food and Drug Administration (FDA) Policy Regarding Sunlamp Products**

FDA promulgated the sunlamp products performance standard, 21 Code of Federal Regulations (CFR) 1040.20, in 1979, 44 Fed. Reg. 65,352 (November 9, 1979), and most recently amended it in 1985, 50 Fed. Reg. 36,548 (September 6, 1985). This regulation requires each sunlamp product to have a label that contains a warning statement with the words:

DANGER **—** Ultraviolet radiation. Follow instructions. Avoid overexposure. As with natural sunlight, overexposure can cause eye and skin injury and allergic reactions. Repeated exposure may cause premature aging of the skin and skin cancer. WEAR PROTECTIVE EYEWEAR; FAILURE TO MAY RESULT IN SEVERE BURNS OR LONG-TERM INJURY TO THE EYES. Medications or cosmetics may increase your sensitivity to the ultraviolet radiation. Consult physician before using sunlamp if you are using medications or have a history of skin problems or believe yourself especially sensitive to sunlight. If you do not tan in the sun, you are unlikely to tan from the use of this product.

21 CFR 1040.20(d)(1)(i). The regulation does not specify requirements for the format in which these words must appear, or the exact location on the product that the warning label must appear, as long as it is "permanently affixed or inscribed on an exterior surface of the product when fully assembled for use so as to be legible and readily accessible to view by the person being exposed immediately before the use of the product." 21 CFR 1040.20(d)(3)(i).

FDA also issued a letter dated June 25, 1985, regarding the warning label to sunlamp product manufacturers outlining FDA policy. The policy letter states

The intended purpose of the warning label required on sunlamp products is to provide that information necessary for the consumer to make an informed decision regarding the risks of using sunlamp products and to provide adequate directions for skin tanning. Therefore, the label must be legible and conspicuously placed on the product so as to render it likely to be read by the user under normal conditions of purchase and use.

Note: The terms "sunlamp products" and "indoor tanning devices" have the same meaning.

Source: FDA (8).

## Methods

In this cross-sectional study, we compiled telephone numbers and addresses from Yellow Pages (for Bronx, Brooklyn, Manhattan, Staten Island, and Queens) and 3 online address sites (Google, Yahoo Local, and Switchboard.com) to determine the number of tanning facilities in New York City, which yielded 183 sites. Of these, telephone outreach identified 85 (46%) tanning facilities. The remaining sites had disconnected telephone numbers or were businesses that did not offer tanning (93 [51%]); 3 (2%) sites offered only spray tanning, and 2 (1%) offered only gel tanning. This process was repeated by a second coder who confirmed that there were 183 tanning facilities listed in New York City.

The observer visited each of the 85 facilities and asked to view the machines that were not occupied by customers. The number viewed at each site varied depending on the number of machines present and available in each site. Direct observations were conducted to assess the number of tanning machines and the presence and visibility of a warning sign posted on each machine observed (Table). All tanning machines were considered, regardless of whether they were beds or stand-up models. Spray tanning devices were found at several facilities studied, and those devices were excluded from our study. All data were collected by a single person (C.H.B.) during April and May 2010. Data analysis involved descriptive statistics, including frequencies and percentages. This study was deemed not human subjects research by the Human Subjects Committee at Columbia University Medical Center.

## Results

Most tanning facilities were in Manhattan (n = 46), followed by Brooklyn, (n = 18), Queens (n = 12), Staten Island (n = 8), and the Bronx (n = 1). Most of the businesses were freestanding tanning facilities (n = 62), although some tanning machines were available in beauty salons (n = 21) and in a fitness facility (n = 2). A variable number of machines were present in each facility (total, 951; mean, 11; range, 1-42), and a different number was viewed in each facility depending on availability (range, 1-8). Of 951 machines, 224 (24%) were observed. Seventy-eight (35%) machines in 47 (55%) of the 85 facilities had no warning labels. For the remaining 146 machines, the warning labels were barely visible (n = 32); moderately visible (n = 54); clearly visible (n = 57); and completely visible (n = 3).

## Discussion

This study was limited by the cross-sectional design, by having only 1 researcher conducting observations and recording data, and by uncertainty about the representativeness of the machines observed. Generalizability of the findings is restricted to New York City. Nevertheless, the findings begin to fill a gap in knowledge regarding compliance with required warnings on tanning machines. No studies were identified that used systematic direct observations of tanning machines to assess the presence and visibility of warnings. One study, conducted more than a decade ago, assessed warnings and other criteria via observation or query of a clerk but did not specify the number of tanning machines observed to measure the presence of warning signs (12).

This study suggests that compliance with federal regulations is low for warning signs on indoor tanning machines in New York City. Research is needed to verify this finding and to assess generalizability to other localities. Warning signs are not sufficient to change consumer behavior (13) but are necessary to help consumers make informed choices about indoor tanning. Regulations requiring posted warnings on tanning machines will not serve their intended purpose if compliance is low, which was found in this study.

## Figures and Tables

**Table:** Categories Used to Code the Visibility of Warning Signs on Tanning Machines in 85 Tanning Facilities, New York City, 2010

**Category**	Characteristics
Not at all visible	No warning sticker or present only in a foreign language.
Barely visible	Warning was present but not very visible because it was placed on the back of a machine, had worn-off print, was only a remnant, or used type of a size and color that made visibility very difficult.
Moderately visible	Difficult to locate the sticker because of odd placement, often on the groove of a stand-up machine between the machine and the door; in bed machines, the sticker was in obscure places on the inside. In all cases, the type was small, making visibility difficult.
Clearly visible	Warning was easier to find, often on the top of a bed machine or on the side of a stand-up machine; the type was easily readable in size, but the text still required effort to read.
Completely visible	Warning was "up front and center." A user would notice it without having to look. On stand-up machines, these would have been affixed to the door and were in large, dark type. On bed machines, these were typically above the latch used to close the machine and were also in large, dark type.
